# Computing scattering resonances using perfectly matched layers with frequency dependent scaling functions

**DOI:** 10.1007/s10543-018-0694-0

**Published:** 2018-01-20

**Authors:** Lothar Nannen, Markus Wess

**Affiliations:** Institut für Analysis und Scientific Computing, TU Wien, Wiedner Hauptstraße 8-10, 1040 Vienna, Austria

**Keywords:** Helmholtz resonance problems, Perfectly matched layer, Frequency dependent complex scaling, Spurious resonances, 65N30, 35J05, 65N25

## Abstract

Using perfectly matched layers for the computation of resonances in open systems typically produces artificial or spurious resonances. We analyze the dependency of these artificial resonances with respect to the discretization parameters and the complex scaling function. In particular, we study the differences between a standard frequency independent complex scaling and a frequency dependent one. While the standard scaling leads to a linear eigenvalue problem, the frequency dependent scaling leads to a polynomial one. Our studies show that the location of artificial resonances is more convenient for the frequency dependent scaling than for a standard scaling. Moreover, the artificial resonances of a frequency dependent scaling are less sensitive to the discretization parameters. Hence, the use of a frequency dependent scaling simplifies the choice of the corresponding discretization parameters.

## Introduction

In this paper, we study acoustic resonances in open systems. In this context an open system is an unbounded domain of wave activity, allowing for the existence of complex resonances (see e.g. [13] for examples). Mathematically speaking, these resonances are the square roots of the eigenvalues of a non self-adjoint operator. Equivalently, they are the poles of a meromorphic extension of a resolvent operator [34].

The real part of a resonance is its frequency and the absolute value of the quotient of its real and imaginary part is the quality factor. A large quality factor makes the solution to the scattering problem very sensitive to external sources in the neighborhood of the resonance frequency (see [13]). Therefore, the computation of scattering resonances is of great interest, not only for acoustic problems, but e.g. for laser cavities and X-ray resonators (see [27]) as well.

A popular numerical approach to such resonance problems is the use of boundary element methods [26, 31]. They are based on the reduction of the problem to the boundary of a given scatterer using the fundamental solution. Since the fundamental solution depends non-linearly on the frequency, this results in non-linear eigenvalue problems. Even though such problems can be solved using the contour integral method [4], for resonance problems it is often more convenient to solve linear eigenvalue problems.

Other methods, including the technique of complex scaling, make use of the decomposition of the unbounded domain into a bounded interior and an unbounded exterior domain. While for the bounded interior problem standard finite elements can be used, the exterior domain needs particular attention because of its unboundedness. Possible ways to treat the problem in the exterior domain include so-called infinite element methods [10, 18] based on a series representation of the solution in the exterior domain, local absorbing boundary conditions [11] and Hardy space infinite element methods [12, 15]. In some cases the latter method is closely related (see [16] for cylindrical waveguide problems) to the complex scaling method, which we are concerned with in the work at hand.

The complex scaling method was introduced amongst others by Simon in the 70s (see [30] or the reviews in [14, 24]) in the context of quantum resonances. Roughly speaking, for this method the real coordinates are stretched into the complex domain such that the imaginary part of the complex coordinates grows at least linearly with increasing distance to the interior domain. Since solutions to the Helmholtz equation on this complex manifold are exponentially decreasing, a truncation of the unbounded exterior domain into a bounded layer leads to an exponentially decreasing modelling error. Now this bounded layer can be discretized using standard finite element methods, which leads to a linear eigenvalue problem.

In 1994 Berenger [2] introduced the popular name perfectly matched layer method for essentially the same method (see [7]). Complex scaling is thereby regarded as an artificial absorbing layer such that no reflections at the interface to the interior domain occur. Convergence of the radial complex scaling for scattering problems was established in [5, 8, 17, 22] under some constraints on the complex damping function. In [3] a truncation free complex scaling was introduced using singular damping functions. Convergence of the radial complex scaling for resonance problems is studied in [20] for homogeneous exterior domains and in [16] for cylindrical waveguide problems.

The main drawback of the complex scaling method is the number of discretization parameters. In order to gain an optimal discretization, the finite element discretizations of the interior and the truncated exterior domain, the thickness of the damping layer, and the slope and the starting point of the damping function have to be adapted to the specific problem. Moreover, the discretization leads to artificial resonances, which can be hard to distinguish from the correct ones (see [19]).

When treating time depending problems with complex scaling, it is common to choose a frequency dependent damping parameter (see e.g. [8]) to guarantee a frequency independent decay of the complex scaled solution. In this paper we extend this idea to resonance problems. Such scalings lead to cubic eigenvalue problems. Therefore, they are more complicated to solve than the linear eigenvalue problems obtained by frequency independent scalings. Nevertheless, the use of a frequency dependent scaling reduces the spurious resonances generated by the discretization of the exterior domain a lot. Therefore, it simplifies the choice of the damping function and the discretization of the exterior domain. To illustrate the behavior of the spurious resonances we present a series of numerical experiments. Moreover, we present ideas on how to categorize and analyze the spurious resonances in the classical, as well as in the frequency dependent case. As a side aspect, we show that spurious resonances are generated already by the discretization of the interior domain, even when the exterior problem is solved exactly.

The remainder of this paper is organized as follows: Radial complex scaling for Helmholtz resonance problems is introduced in Sect. 2. In Sect. 3 we formulate the corresponding eigenvalue problems. Section 4 contains numerical studies in a simple one-dimensional setting. Two-dimensional problems are studied in Sect. 5, and a short conclusion completes the paper.

## Settings

We study the dependency of artificial resonances on two different types of radial complex scalings. Namely a frequency independent linear complex scaling, and a frequency dependent one.

### The resonance problem

Let $\varOmega \subset \mathbb {R}^{d}$ with $d=1,2$ be an unbounded Lipschitz domain and $R>0$ such that ${\varOmega _\mathrm{ext}}:=\{\mathbf {x} \in \mathbb {R}^d: \Vert \mathbf {x}\Vert >R\}\subset \varOmega$ and $\partial {\varOmega _\mathrm{ext}}\cap \partial \varOmega =\emptyset$. We separate $\varOmega$ into the unbounded exterior domain ${\varOmega _\mathrm{ext}}$, the bounded interior domain ${\varOmega _\mathrm{int}}:=\varOmega {\setminus }\overline{{\varOmega _\mathrm{ext}}}$ and the interface $\varGamma :=\partial {\varOmega _\mathrm{ext}}$, i.e. $\varOmega ={\varOmega _\mathrm{int}}\cup \varGamma \cup {\varOmega _\mathrm{ext}}$.

Moreover, let $p\in L^\infty (\varOmega )$ be an almost everywhere positive potential with $p\equiv 1$ in $\overline{{\varOmega _\mathrm{ext}}}$. We are looking for eigenpairs $(\omega ,u)\in \mathbb {C}\times H^1({\varOmega _\mathrm{int}}){\setminus }\{0\}$ with $\mathfrak {R}(\omega )>0$ such that

2.1a $$\begin{aligned} -\varDelta u&= \omega ^2 p\, u&\quad \text {in } \quad \varOmega , \end{aligned}$$

2.1b $$\begin{aligned} {{\mathrm{\mathrm {B}}}}u&= 0&\quad \text {on } \quad \partial \varOmega , \end{aligned}$$

2.1c $$\begin{aligned}&u \text { is outgoing}&\quad \text {for} \quad \Vert \mathbf {x}\Vert \rightarrow \infty . \end{aligned}$$

${{\mathrm{\mathrm {B}}}}$ is a trace operator on $\partial \varOmega$, e.g. a Dirichlet trace operator ${{\mathrm{\mathrm {B}}}}u:=u|_{\partial \varOmega }$ or a Neumann trace operator ${{\mathrm{\mathrm {B}}}}u:=\nabla u \cdot \mathbf {n}|_{\partial \varOmega }$ with normal vector $\mathbf {n}$. There exist several different formulations for the radiation condition (2.1c). For resonance problems, typically a representation of the form

2.2 $$\begin{aligned} u(\mathbf {x})={\left\{ \begin{array}{ll} c \exp (\mathfrak {i}\omega | x|),\quad &{}d=1\\ \sum \nolimits _{\nu =-\infty }^\infty c_\nu H_{|\nu |}^{(1)}(\omega \Vert \mathbf {x}\Vert ) \varPhi _\nu \left( \frac{\mathbf {x}}{\Vert \mathbf {x}\Vert }\right) , \quad &{}d=2, \end{array}\right. },\qquad \mathbf {x}\in {\varOmega _\mathrm{ext}}, \end{aligned}$$

with $c,c_\nu \in \mathbb {C}$, $\nu \in \mathbb {Z}$ is used. The symbol $\varPhi _n$ denotes the two-dimensional spherical harmonic and $H_{n}^{(1)}$ the Hankel function of the first kind of index *n*. For the details we refer to [9]. There it is shown that the series, as well as its term by term derivatives converge uniformly on a suitable compact domain. Moreover, due to [23, Eq. (5.11.4)] it holds

2.3 $$\begin{aligned} H_\nu ^{(1)}(r)=\sqrt{\frac{2}{\pi r}} \exp \left( \mathfrak {i}\left( r-\nu \frac{\pi }{2}-\frac{\pi }{4}\right) \right) \left( 1+{{\mathrm{\mathcal{O}}}}\left( \frac{1}{r}\right) \right) ,\text { for } |r| \rightarrow \infty , \end{aligned}$$

in $\mathbb {C}{\setminus }\mathbb {R}_{\le 0}$. Hence, *u* given by (2.2) increases exponentially towards infinity for frequencies $\omega$ with negative imaginary part.

#### Remark 2.1

A mathematically correct framework for this kind of resonance problems is the theory of holomorphic Fredholm operator functions (see e.g. [32, §9]). Based on reformulations of (2.1) by integral equations it is shown that there exists only a discrete set of resonances, for $p\equiv 1$. These resonances have positive real and negative imaginary parts. In particular, for problems where $\varOmega$ is the complement of a sphere with Dirichlet boundary conditions, the resonances are the scaled roots of the Hankel functions. There exist $|\nu |$ roots with positive real and negative imaginary part (see [1, p. 373]).

### Complex scaling

In principle, most complex scaling methods are composed of three steps:

- formulation of a complex scaled resonance problem [see (2.8)],
- truncation of $\varOmega$ to a bounded domain $\varOmega _T$ [see (2.9)], and
- finite element discretization of $H^1(\varOmega _T)$ [see (2.10)].

Note that the second step is not necessary for complex scaling methods with singular damping profiles (see e.g. [3]). Starting with the first step, we choose $R>0$ and $\sigma \in \mathbb {C}$ with $\mathfrak {R}(\sigma ),\,\mathfrak {I}(\sigma )>0$ and define the continuous damping function $\gamma _{\sigma ,R}:\mathbb {R}_{\ge 0} \rightarrow \mathbb {C}$ by

2.4 $$\begin{aligned} \gamma _{\sigma ,R}(r):={\left\{ \begin{array}{ll} r,&{}\quad r\le R\\ \sigma (r-R)+R, &{}\quad r>R \end{array}\right. }. \end{aligned}$$

In the following we will use two different types of this damping. In a standard linear complex scaling $\sigma$ is constant while for a frequency dependent complex scaling we use $\sigma (\omega )=\sigma _0/\omega$ with constant $\sigma _0\in \mathbb {C}$. Note that this frequency dependent complex scaling in the real part differs from the scaling $\sigma (\omega )=\mathfrak {R}(\sigma _0)-\mathfrak {I}(\sigma _0)/(\mathfrak {i}\omega )$ used for time-dependent problems (see e.g. [8]).

Based on this damping function, we introduce a complex scaled variable

2.5 $$\begin{aligned} {\hat{\mathbf {x}}}_{\sigma ,R}(\mathbf {x}):={\left\{ \begin{array}{ll} \frac{\gamma _{\sigma ,R}(\Vert \mathbf {x}\Vert )}{\Vert \mathbf {x}\Vert } \mathbf {x}, &{}\quad \mathbf {x}\ne 0\\ 0, &{}\quad \mathbf {x} = 0 \end{array}\right. }, \end{aligned}$$

for $\mathbf {x} \in \varOmega \subset \mathbb {R}^d$. Since $\mathfrak {I}(\sigma )>0$, we have ${\hat{\mathbf {x}}}_{\sigma ,R} \in \{ z \in \mathbb {C}:\mathfrak {I}(z)>0\}^d$ for $\Vert \mathbf {x}\Vert >R$ and ${\hat{\mathbf {x}}}_{\sigma ,R}=\mathbf {x}$ for $\Vert \mathbf {x}\Vert \le R$. For all $\Vert \mathbf {x}\Vert \ne R$ the function $\gamma _{\sigma ,R}$ is arbitrarily smooth with Jacobian $J_{\sigma ,R}(\mathbf {x}):=\frac{\partial }{\partial \mathbf {x}} {\hat{\mathbf {x}}}_{\sigma ,R}(\mathbf {x})$, which depends on $\omega$ in the frequency dependent case.

For a resonance function *u* of (2.1) we define the complex scaled function $\hat{u}_{\sigma ,R}$ by

2.6 $$\begin{aligned} \hat{u}_{\sigma ,R}:= u \circ {\hat{\mathbf {x}}}_{\sigma ,R}. \end{aligned}$$

Using the techniques in [9], the mappings $r\mapsto u|_{{\varOmega _\mathrm{ext}}}(r \hat{\mathbf {x}})$ with $\hat{\mathbf {x}}:=\mathbf {x}/\Vert \mathbf {x}\Vert$ and $u|_{{\varOmega _\mathrm{ext}}}$ given by (2.2) can be holomorphically extended to $\mathbb {C}{\setminus } \mathbb {R}_{\le 0}$. Hence, $\hat{u}_{\sigma ,R}$ is well defined. Due to the asymptotic behavior (2.3) of the Hankel functions, $\hat{u}_{\sigma ,R}$ decays exponentially if and only if

2.7 $$\begin{aligned} \mathfrak {I}(\omega \sigma )>0, \end{aligned}$$

since due to [6, Lem. 4.3] the exponential decay of the scaled Hankel functions is uniform in the index $\nu$. For a frequency dependent scaling $\sigma (\omega )=\sigma _0/\omega$ (2.7) is always ensured if $\mathfrak {I}(\sigma _0)>0$. For a frequency independent scaling and a given resonance $\omega$ with $\mathfrak {I}(\omega )<0$ (2.7) is only satisfied if the damping $\mathfrak {I}(\sigma )/\mathfrak {R}(\sigma )$ is sufficiently large such that the exponential increase of the according resonance function is annihilated. This is a first indicator, that the frequency dependent scaling differs substantially from a standard complex scaling. Let $(\omega ,u_\mathrm{int})\in \mathbb {C}\times H^1({\varOmega _\mathrm{int}}){\setminus }\{0\}$ be an eigenpair of (2.1) and let $\hat{u}_{\sigma ,R}$ be the complex scaled eigenfunction as in (2.6) such that (2.7) holds. Then $(\omega ,\hat{u}_{\sigma ,R}) \in \mathbb {C}\times V$ is an eigenpair of the complex scaled resonance problem

2.8 $$\begin{aligned} \int _\varOmega J_{\sigma ,R}^{-T} \nabla u \cdot J_{\sigma ,R}^{-T} \nabla v \,\det (J_{\sigma ,R}) \,d\mathbf {x} = \omega ^2 \int _\varOmega p\, u \, v \, \det (J_{\sigma ,R}) \,d\mathbf {x},\quad v\in V. \end{aligned}$$

For Neumann boundary conditions at $\partial \varOmega$ we use $V=H^1(\varOmega )$. Dirichlet boundary conditions have to be incorporated into *V* as usual. Note that the complex scaled resonance function $\hat{u}_{\sigma ,R}$ is, at least, not smooth for $\{\mathbf {x} \in \mathbb {R}^d:\Vert \mathbf {x}\Vert =R\}$ since the scaling $\gamma _{\sigma ,R}$ defined in (2.4) is not smooth there.

#### Remark 2.2

Instead of a radial complex scaling (2.5), commonly a Cartesian complex scaling is used. In this case the damping function defined in (2.4) is applied to each Cartesian component of $\mathbf {x}$ separately. Typically, for these scalings the exterior domain is the complement of a cube, i.e. ${\varOmega _\mathrm{ext}}=\mathbb {R}^d{\setminus } [-R,R]^d$. We refer to [21] for such a scaling for resonance problems. In principle, the frequency dependent complex scaling used in this paper can be applied to Cartesian scalings as well. Nevertheless, we confined ourselves to radial scalings since the study of artificial resonances is easier in this case. For the same reasons we only use a linear complex scaling in (2.4). A damping profile $\sigma =\sigma (r)$ with smooth function $\sigma :\mathbb {R}_{\ge 0} \rightarrow \mathbb {C}$ could be combined with a frequency dependent scaling as well.

### Discretization and truncation

Problem (2.8) is still posed on the unbounded domain $\varOmega$ and therefore not directly feasible for a finite element discretization. Thus, $\varOmega$ is typically truncated to a finite domain $\varOmega _T:=\{\mathbf {x} \in \varOmega : \Vert \mathbf {x}\Vert \le T\}$. If (2.7) holds, the resonance functions $\hat{u}_{\sigma ,R}$ constructed from eigenpairs of (2.1) decay exponentially, and we expect the truncation error to be small if $T>R$ is large enough. Using homogeneous Neumann boundary conditions at the truncation boundary $\partial \varOmega _T{\setminus } \partial \varOmega$, we are looking for eigenpairs $(\omega _T,\hat{u}_{\sigma ,R,T}) \in \mathbb {C}\times V_T$ of

2.9 $$\begin{aligned} \int _{\varOmega _T} J_{\sigma ,R}^{-T} \nabla u \cdot J_{\sigma ,R}^{-T} \nabla v \,\det (J_{\sigma ,R}) \,d\mathbf {x} = \omega ^2 \int _{\varOmega _T} p\, u \, v \, \det (J_{\sigma ,R}) \,d\mathbf {x},\quad v\in V_T, \end{aligned}$$

with $$V_T:=H^1(\varOmega _T)$$.

In the following, we study the different effects of the finite element discretization. Let $V_\mathrm{int}^h \subset H^1({\varOmega _\mathrm{int}})$ and $V_\mathrm{ext}^h \subset H^1(\varOmega _T{\setminus } {\varOmega _\mathrm{int}})$ be constructed such that the trace spaces on the interface $\varGamma =\overline{{\varOmega _\mathrm{int}}}\cap \overline{{\varOmega _\mathrm{ext}}}$ coincide, i.e. $\{f|_\varGamma :f\in V_\mathrm{int}^h\}=\{f|_\varGamma :f\in V_\mathrm{ext}^h\}$. We solve for eigenpairs $( \omega _{h_i,h_e},u_{h_i,h_e})\in \mathbb {C}\times V_{h_i,h_e}$ of

2.10 $$\begin{aligned} \int _{\varOmega _T} J_{\sigma ,R}^{-T} \nabla u \cdot J_{\sigma ,R}^{-T} \nabla v \,\det (J_{\sigma ,R}) \,d\mathbf {x} = \omega ^2 \int _{\varOmega _T} p\, u \, v \, \det (J_{\sigma ,R}) \,d\mathbf {x},\quad v\in V_{h_i,h_e}, \end{aligned}$$

with

2.11 $$\begin{aligned} V_{h_i,h_e}:=\left\{ f\in V_T: f|_{{\varOmega _\mathrm{int}}}\in V_\mathrm{int}^{h_i} \wedge f|_{\varOmega _T{\setminus } {\varOmega _\mathrm{int}}}\in V_\mathrm{ext}^{h_e}\right\} , \end{aligned}$$

and $$\mathfrak {R}(\omega _{h_i,h_e})>0$$, $$u_{h_i,h_e}\ne 0$$.

In our numerical tests we choose different parameters $T,h_i$ and $h_e$ to show the different discretization effects. In particular we study the effects of

D.a discretization of the interior domain (Sect. 4.1),
D.b truncation (Sect. 4.2),
D.c discretization of the interior domain and truncation (Sect. 4.3),
D.d discretization of the truncated exterior domain (Sect. 4.4), and
D.e full discretization and truncation (Sects. 4.5 and 5).

With these numerical tests we pursue two objectives: First we classify the different types of artificial resonances and their dependency on the discretization parameters. To potential users of the frequency independent, as well as the frequency dependent complex scaling, these studies should be helpful in choosing appropriate parameters. Second we show how a frequency dependent scaling affects the artificial resonances. It turns out that a frequency dependent scaling might be particularly useful for resonances with large real and small imaginary parts.

## The frequency dependent complex scaled Helmholtz problem

In this section we deal with the (non-linear) eigenvalue problems arising from (2.8) and the use of frequency dependent scaling functions $\sigma \left( \omega \right)$.

### The essential spectrum

#### Definition 3.1

For some open set $\varLambda \subset \mathbb {C}$, and some Hilbert space *X*, let $T\left( \omega \right) :D\subset X\rightarrow X$ be a densely defined linear operator, for each $\omega \in \varLambda$. Following [14], we define

3.1 $$\begin{aligned} \varSigma \left( T\right) :=\left\{ \omega \in \varLambda :T\left( \omega \right) \text { is not boundedly invertible}\right\} . \end{aligned}$$

We decompose $\varSigma \left( T\right)$ into two disjoint subsets

3.2 $$\begin{aligned} \varSigma \left( T\right)&=\varSigma _{ess}\left( T\right) \dot{\cup }\varSigma _d\left( T\right) , \end{aligned}$$

where $\varSigma _d\left( T\right)$ is the set of isolated eigenvalues with finite algebraic multiplicity.

Let $L_{\sigma ,R}\left( \omega \right)$ be the operator defined weakly by

3.3 $$\begin{aligned} \int _\varOmega \left( J_{\sigma ,R}^{-T}\nabla u\cdot J_{\sigma ,R}^{-T}\nabla v-\omega ^2p u v \right) \det J_{\sigma ,R}\,d\mathbf {x} = \int _\varOmega L_{\sigma ,R}\left( \omega \right) u\, v \,d\mathbf {x}, \end{aligned}$$

with domain ${{\mathrm{\mathrm {dom}}}}\left( L_{\sigma ,R}\left( \omega \right) \right) :=\{u\in L^2(\varOmega ): L_{\sigma ,R}\left( \omega \right) u \in L^2(\varOmega )\}$. Then $L_{\sigma ,R}\left( \omega \right) :{{\mathrm{\mathrm {dom}}}}\left( L_{\sigma ,R}\left( \omega \right) \right) \subset L^2\left( \varOmega \right) \rightarrow L^2\left( \varOmega \right)$ is an (unbounded) operator, and (2.8) is a weak form of the eigenvalue problem $L_{\sigma ,R}\left( \omega \right) u=0$.

For a frequency independent scaling $\sigma (\omega )\equiv \sigma _0$, it is shown in [14] that $\varSigma _{ess}\left( L_{\sigma ,R}\right) =\left\{ \omega \in \mathbb {C}:\arg \omega ^2+2\arg \sigma =0\right\}$ for $\varOmega =\mathbb {R}^n$. Restricted to frequencies $\omega$ with positive real part this is equivalent to $\mathfrak {I}(\sigma \omega )=0$ (confer with (2.7)).

By a similar reasoning as in [21, Thm. 4.6], one can show that the essential spectrum does not change for $\varOmega =\mathbb {R}^n{\setminus } \varOmega _0$ and compact $\varOmega _0$. By the same reasoning, or alternatively by using compact perturbation theory, one can see that $\varSigma _{ess}\left( L_{\sigma ,R}\right)$ is independent of the potential function *p* as well since *p* has compact support by assumption.

For a frequency dependent scaling of the form $\sigma (\omega )=\sigma _0/\omega$ with $\mathfrak {I}(\sigma _0)>0$, $L_{\sigma ,R}\left( \omega \right)$ is, in contrast to a frequency independent scaling, non-linear in $\omega ^2$. Nevertheless, we can use the preceding results for the linear eigenvalue problem to show that for $\omega \ne 0$ the operator $L_{\sigma ,R}\left( \omega \right)$ is either invertible or $\omega$ is an eigenvalue, since $\mathfrak {I}(\sigma (\omega )\omega )=\mathfrak {I}(\sigma _0)>0$. In other words for $\sigma =\sigma _0/\omega$ there is no essential spectrum with positive real part.

### Solving the non-linear eigenvalue problem

Plugging finite element basis functions into problem (2.10) for frequency dependent $\sigma \left( \omega \right)$ leads to a discrete eigenvalue problem of the form $M\left( \omega \right) =0$, where $M:\mathbb {C}\rightarrow \mathbb {C}^{k\times k}$ is a matrix valued function. Available numerical methods for treating discrete non-linear eigenvalue problems include the contour integral method for analytic *M* (see e.g. [4]) and the reduction to a linear eigenvalue problem by substitution for rational problems of the form

3.4 $$\begin{aligned} 0=M\left( \omega \right) =\sum _{j=N_0}^{N_1} M_j\omega ^j. \end{aligned}$$

Here $M_j\in \mathbb {C}^{k\times k}$ are coefficient matrices and $N_0<N_1\in \mathbb {Z}$. Unfortunately, for a frequency dependent complex scaling the discrete formulation (2.10) in two dimensions in general is not of the form (3.4). This, makes a straightforward reduction to a linear eigenvalue problem impossible. Even if the contour integral method makes the treatment of such an eigenvalue problem possible, it is still advantageous to apply it to rational eigenvalue problemssince the evaluation of $M\left( \omega \right)$ is less expensive.

To obtain an eigenvalue problem of the form (3.4), we use test functions of the form

3.5 $$\begin{aligned} \tilde{v}\left( \mathbf {x}\right) := {\left\{ \begin{array}{ll} v\left( \mathbf {x}\right) ,&{} \quad \mathbf {x} \in {\varOmega _\mathrm{int}},\\ \frac{\gamma _{\sigma ,R}\left( \Vert \mathbf {x}\Vert \right) }{R}v\left( \mathbf {x}\right) ,&{}\quad \mathbf {x}\in {\varOmega _\mathrm{pml}}:={\varOmega _\mathrm{ext}}\cap \varOmega _T. \end{array}\right. } \end{aligned}$$

Since $0\notin {\varOmega _\mathrm{ext}}$, the map defined by (3.5) is an automorphism on $H^1\left( \varOmega _T\right)$.

Plugging test functions $\tilde{v}$ into (2.9) gives after some calculations

3.6 $$\begin{aligned} A_{\mathrm {int}}\left( u,v\right) +A_{\mathrm {pml}}\left( u,v\right) =\omega ^2 \left( B_{\mathrm {int}}\left( u,v\right) +B_{\mathrm {pml}}\left( u,v\right) \right) , \end{aligned}$$

with

3.7 $$\begin{aligned} A_{\mathrm {int}}\left( u,v\right) :=\int _{{\varOmega _\mathrm{int}}}\nabla u\cdot \nabla v\,d\mathbf {x},\quad B_{\mathrm {int}}\left( u,v\right) :=\int _{{\varOmega _\mathrm{int}}}puv\,d\mathbf {x}, \end{aligned}$$

and

3.8a $$\begin{aligned} A_{\mathrm {pml}}\left( u,v\right) :={}&\frac{1}{R}\int _{{\varOmega _\mathrm{pml}}}\gamma '_{\sigma ,R}\left( \Vert \mathbf {x}\Vert \right) \Vert \mathbf {x}\Vert \nabla u\cdot \nabla v+\frac{\gamma _{\sigma ,R}\left( \Vert \mathbf {x}\Vert \right) }{\Vert \mathbf {x}\Vert ^2}\nabla u\cdot \mathbf {x} v\nonumber \\&+\left( \frac{\gamma ^2_{\sigma ,R}\left( \Vert \mathbf {x}\Vert \right) }{\gamma '_{\sigma ,R}\left( \Vert \mathbf {x}\Vert \right) \Vert \mathbf {x}\Vert ^3}-\frac{\gamma _{\sigma ,R}'\left( \Vert \mathbf {x}\Vert \right) }{\Vert \mathbf {x}\Vert }\right) \nabla u\cdot \mathbf {x}\mathbf {x}^T\nabla v\,d\mathbf {x}\end{aligned}$$

3.8b $$\begin{aligned} B_{\mathrm {pml}}\left( u,v\right) :={}&\int _{{\varOmega _\mathrm{pml}}}uv\frac{\gamma ^2_{\sigma ,R}\left( \Vert \mathbf {x}\Vert \right) \gamma '_{\sigma ,R}\left( \Vert \mathbf {x}\Vert \right) }{R\Vert \mathbf {x}\Vert }\,d\mathbf {x}. \end{aligned}$$

For a damping function $\gamma _{\sigma ,R}\left( r\right) :=\frac{\sigma _0}{\omega }\left( r-R\right) +R$, we obtain a rational eigenvalue problem of the form $\sum _{j=-1}^2\omega ^j A_j\left( u,v\right) =0$ with bilinear forms

3.9a $$\begin{aligned} A_{-1}\left( u,v\right) :={}&\frac{\sigma _0}{R}\int _{{\varOmega _\mathrm{pml}}}\Vert \mathbf {x}\Vert \nabla u\cdot \nabla v\,d\mathbf {x} +\frac{\sigma _0}{R}\int _{\varOmega _\mathrm{pml}}\frac{\Vert \mathbf {x}\Vert -R}{\Vert \mathbf {x}\Vert ^2}\mathbf {x}\cdot \nabla u\,v\,d\mathbf {x} \nonumber \\&+\frac{\sigma _0}{R}\int _{\varOmega _\mathrm{pml}}\left( \frac{\left( \Vert \mathbf {x}\Vert -R\right) ^2}{\Vert \mathbf {x}\Vert ^3}-\frac{1}{\Vert \mathbf {x}\Vert }\right) \nabla u\cdot \mathbf {x}\mathbf {x}^T\nabla v\,d\mathbf {x} \nonumber \\&-\frac{\sigma _0^3}{R}\int _{\varOmega _\mathrm{pml}}\frac{\left( \Vert \mathbf {x}\Vert -R\right) ^2}{\Vert \mathbf {x}\Vert }uv\,d\mathbf {x},\end{aligned}$$

3.9b $$\begin{aligned} A_{0}\left( u,v\right) :={}&\int _{\varOmega _\mathrm{int}}\nabla u\cdot \nabla v\,d\mathbf {x}+2\int _{\varOmega _\mathrm{pml}}\frac{\Vert \mathbf {x}\Vert -R}{\Vert \mathbf {x}\Vert ^3}\nabla u\cdot \mathbf {x}\mathbf {x}^T\nabla v\,d\mathbf {x} \nonumber \\&+\int _{\varOmega _\mathrm{pml}}\frac{1}{\Vert \mathbf {x}\Vert ^2}\mathbf {x}\cdot \nabla u\,v\,d\mathbf {x}-2\sigma _0^2\int _{\varOmega _\mathrm{pml}}\frac{\Vert \mathbf {x}\Vert -R}{\Vert \mathbf {x}\Vert }uv\,d\mathbf {x},\end{aligned}$$

3.9c $$\begin{aligned} A_{1}\left( u,v\right) :={}&\frac{R}{\sigma _0}\int _{\varOmega _\mathrm{pml}}\frac{1}{\Vert \mathbf {x}\Vert ^3}\nabla u\cdot \mathbf {x}\mathbf {x}^T\nabla v\,d\mathbf {x}-R\sigma _0\int _{\varOmega _\mathrm{pml}}\frac{1}{\Vert \mathbf {x}\Vert }uv\,d\mathbf {x},\end{aligned}$$

3.9d $$\begin{aligned} A_{2}\left( u,v\right) :={}&-\int _{\varOmega _\mathrm{int}}puv\,d\mathbf {x}. \end{aligned}$$

Plugging in discrete test and ansatz functions results in a rational eigenvalue problem (3.4) with matrices $M_j$ associated with the respective bilinear forms (3.9).

#### Remark 3.1

Using a cartesian scaling (cf. Remark 2.2) with a frequency dependent damping function would lead to a rational eigenvalue problem (3.4) immediately.

## Effects of discretization in one dimension

In this section we study the effects of truncating and discretizing the complex scaled problem by comparing the frequency dependent and the frequency independent scaling in one dimension.

Let ${\varOmega _\mathrm{int}}:=\left( 0,R\right)$, $p_0\ge 1$ and $R\ge R_0>0$ be given. We define the potential function by $p\left( x\right) :=p_0^2\chi _{\left[ 0,R_0\right] }\left( x\right)$, where $\chi _M$ denotes the characteristic function of a set *M*. Moreover, we use Neumann boundary conditions at $\partial \varOmega$, i.e. $u'(0)=0$. Using the ansatz

4.1 $$\begin{aligned} u\left( x\right) = {\left\{ \begin{array}{ll}C_1\exp \left( -ip_0\omega x\right) +C_2\exp \left( ip_0\omega x\right) ,&{} \quad x\in [0,R_0){,}\\ C_3\exp \left( -i\omega x\right) +C_4\exp \left( i\omega x\right) ,&{} \quad x\in [R_0,R], \end{array}\right. } \end{aligned}$$

the radiation condition (2.2) and regularity of *u* at $R_0$, straightforward calculations show that the resonances of this one-dimensional problem are given by

4.2 $$\begin{aligned} \omega _k:=\frac{1}{2p_0R_0}\left( -i\log \left( \frac{p_0+1}{p_0-1}\right) +2k\pi \right) ,\quad k\in \mathbb Z, \end{aligned}$$

for $p_0>1$. There are no resonances for $p_0=1$.

### Discretization of the interior domain

Prior to dealing with the effects of the complex scaling on the discrete spectrum, we present a short analysis of the effects of discretizing the interior domain only. This is essential for the following examination of the complex scaled problem. In one dimension this can be easily done by using a finite element approximation in ${\varOmega _\mathrm{int}}$ and the correct radiation condition realized by the Robin boundary condition $u'(R)=i\omega u(R)$. All of the following results were obtained using finite element basis functions of polynomial order 6 with a uniform mesh-size *h*.

**Fig. 1:**
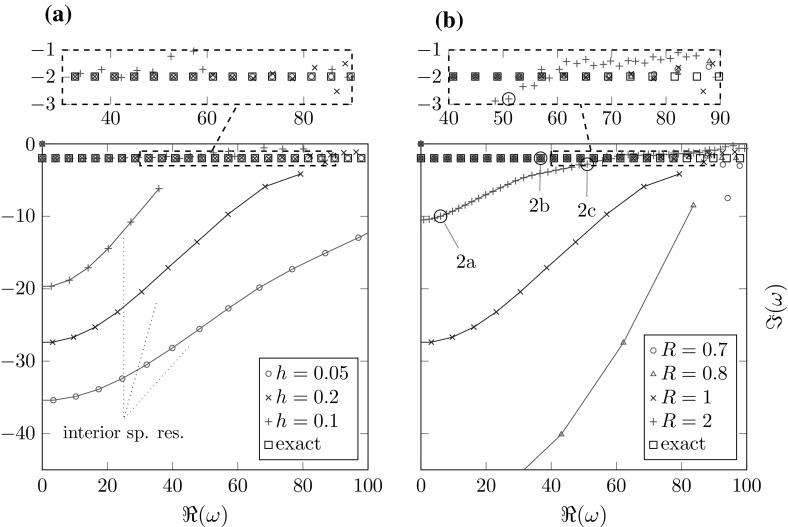
Resonances of the one-dimensional problem using the exact radiation condition. **a** Fixed potential $p_0=1.1$, $R_0=0.7$ and boundary $R=1$. **b** Fixed mesh-size $h=0.1$ and potential $p_0=1.1$, $R_0=0.7$

**Fig. 2:**
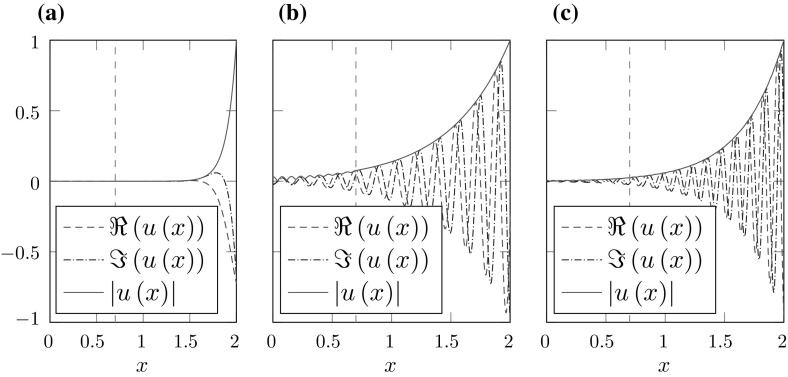
Eigenfunctions *u* in $\varOmega _{int}$ corresponding to selected resonances $\omega$ from Fig. 1b. The vertical dashed line marks the jump in the potential function *p*. **a** $\omega \approx 6.0193{-}9.9883i$, **b** $\omega \approx 36.7223{-}1.9790i$, **c** $\omega \approx 51.0383{-}2.8031i$

Figure 1a shows the approximated resonances for different mesh-sizes *h*. The calculations exhibit a set of spurious resonances generated by the discretization, where the approximation quality of the true resonances corresponds roughly with their distance to these resonances. For smaller mesh-sizes *h* the curve connecting these resonances moves farther away from the real axis.

In Fig. 1b the mesh-size *h* is fixed while the parameter *R* is varied. Note that for $R=R_0$ the spurious resonances vanish. The larger $R-R_0$ gets, the less correct eigenvalues are calculated. This behavior is a consequence of the fact that the eigenfunctions increase exponentially in ${\varOmega _\mathrm{int}}$ (cf. Fig. 2). Therefore, discretization errors generated in $[0,R_0]$ are amplified with increasing distance $R-R_0$.

**Fig. 3:**
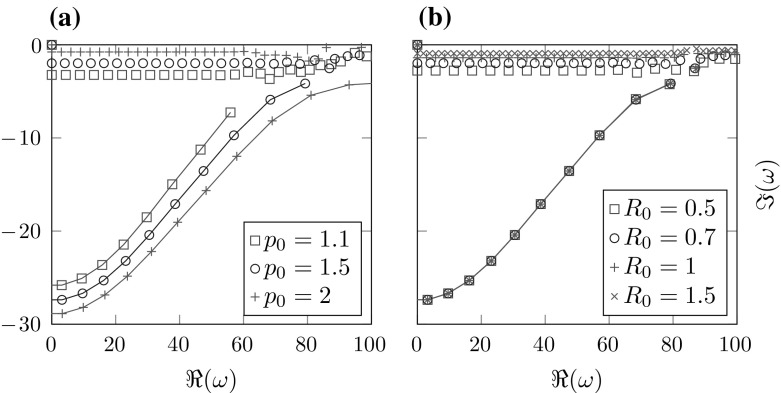
Resonances for a fixed mesh-size $h=0.1$, different potentials $p_0$, $R_0$ and boundaries *R*. **a** $R_0=0.7$, $R=1$, **b** $R=R_0+0.3$, $p_0=1.1$

Taking into account Fig. 3b, we can conclude that, besides the mesh-size *h*, the relevant quantity for the location of the spurious resonances is indeed the distance of the jump in the potential function to the boundary of the interior domain $R-R_0$. Figure 2 shows three eigenfunctions corresponding to eigenvalues from Fig. 1b. While 2b is a correct resonance, 2a, c correspond to spurious resonances. Note that distinguishing the spurious from the correct resonances by the shape of the eigenfunctions seems to be impossible in this example.

All in all we can conclude that, even in this simple example, spurious resonances are generated by the discretization of the interior domain already if $R-R_0>0$. Their location depends heavily on $R-R_0$ and *h* (cf. Figs. 1a, b and 3b) and to a lesser extent on the potential parameter $p_0$ (cf. Fig. 3a). In the following we will refer to these spurious resonances as *interior spurious resonances*.

### Truncation

Let $T>R$ and $\varOmega _T:=\left( 0,T\right) ={\varOmega _\mathrm{int}}\cup \varGamma \cup \varOmega _{\mathrm {pml}}$, where ${\varOmega _\mathrm{int}}:=\left( 0,R\right)$, $\varGamma :=\{R\}$, and $\varOmega _{\mathrm {pml}}:=\left( R,T\right)$. If we prescribe Neumann boundary conditions at $\{0,T\}$, the resonances of (2.9) can be calculated using a similar ansatz to (4.1) and satisfy

4.3 $$\begin{aligned} p_0\tan \left( p_0R\omega \right) +\tan \left( (T-R)\sigma \omega \right) =0. \end{aligned}$$

Using a frequency dependent and a frequency independent scaling of the form (2.4) with damping parameters $\sigma _1\left( \omega \right) :\equiv \sigma _0$, $\sigma _2\left( \omega \right) :=\frac{\sigma _0}{\omega }$, $\sigma _0\in \mathbb {C}$, $\mathfrak {I}\left( \sigma _0\right) >0$, leads to resonances

4.4a $$\begin{aligned} \omega _{k,T}^{\sigma _1}&=\frac{\frac{k\pi }{T}}{\frac{R(1-\sigma _0)}{T}+\sigma _0},&\quad k\in \mathbb Z,\end{aligned}$$

4.4b $$\begin{aligned} \omega _{k,T}^{\sigma _2}&=-\frac{\sigma _0(1-\frac{R}{T})+\frac{k\pi }{T}}{\frac{R}{T}},&\quad k\in \mathbb Z, \end{aligned}$$

respectively, for $p_0=1$ (recall that in this case there are no correct resonances). The resonances (4.4a) are a discretization of the essential spectrum since $\{\omega _{k,T}^{\sigma _1}:k\in \mathbb Z\}\rightarrow \{\omega \in \mathbb {C}:\arg \left( \omega \sigma _0\right) =0\}$ for $T\rightarrow \infty$ (cf. 3.1). The resonances (4.4b) diverge for $T\rightarrow \infty$. Both of them will be referred to as *truncation resonances*. Note that $\mathfrak {I}\left( \omega _{k,T}^{\sigma _2}\right) =-\mathfrak {I}(\sigma _0)\left( \frac{T}{R}-1\right) <0$ is constant for all $k\in \mathbb Z$. This corresponds to the fact that the decay of the complex scaled eigenfunctions in the frequency dependent case is independent of the frequency (cf. Fig. 6) since this decay depends on the imaginary part of the eigenvalues. By the implicit function theorem, we can conclude that this behavior of the truncation resonances is also valid for $p_0\approx 1$, although in this case there are additional correct resonances of (2.1).

**Fig. 4:**
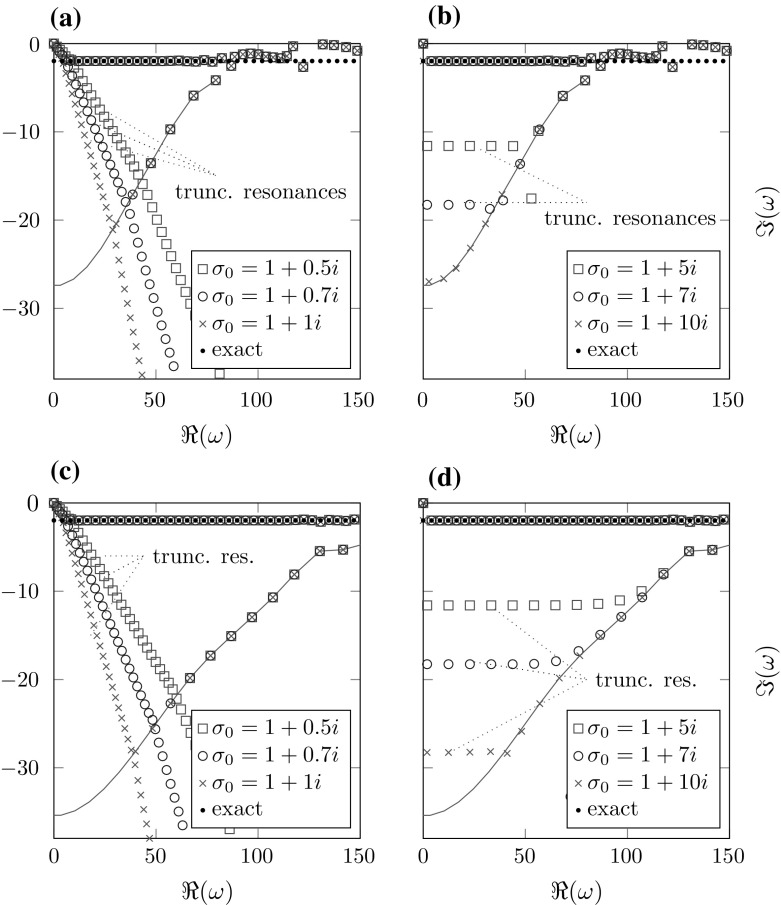
Resonances for different complex scalings with a very fine exterior discretization. The teal line marks the location of the interior spurious resonances calculated using the exact radiation condition (cf. Sect. 4.1). **a** $\sigma \equiv \sigma _0$, $h_\mathrm{int}=0.1$, **b** $\sigma =\frac{\sigma _0}{\omega }$, $h_\mathrm{int}=0.1$, **c** $\sigma \equiv \sigma _0$, $h_\mathrm{int}=0.05$, **d** $\sigma =\frac{\sigma _0}{\omega }$, $h_\mathrm{int}=0.05$

### Discretization of the interior domain and truncation

We study the effects of discretizing the interior domain and truncation using basis functions of order 10 in the exterior domain and of order 6 in the interior domain. Figure 4 shows the effects of varying different parameters for scalings $\sigma _1,\sigma _2$ as defined in Sect. 4.2.

Two kinds of artificial resonances can be observed: The truncation resonances from (4.4a) and (4.4b) and the interior spurious resonances (cf. Sect. 4.1). In both the frequency dependent and the frequency independent case we only see interior spurious resonances located above the line containing the truncation resonances.

Note that the interior spurious resonances are almost independent of the type of scaling and the parameters $\sigma _0,\,T-R$. They are merely a result of the discretization of the interior domain. As indicated by (4.4b), the truncation resonances in the frequency dependent case move farther away from the real axis for growing $\mathfrak {I}(\sigma _0)$. For the frequency independent scaling we see almost straight rays starting from the origin and depending on $\arg (\sigma _0)$ as expected by (4.4a).

**Fig. 5:**
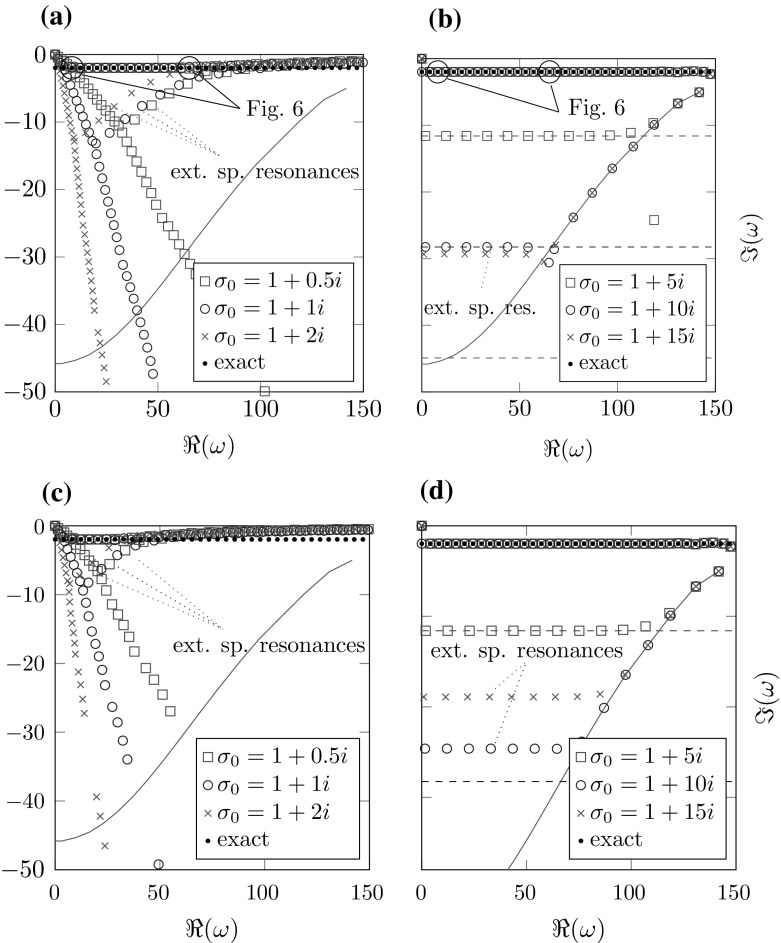
Resonances for different complex scalings with coarse exterior discretization. The teal line marks the location of the interior spurious resonances (cf. Sect. 4.1). The horizontal dashed lines in the frequency scaled case show the locations of the truncation resonances (cf. Sect. 4.2). **a** $\sigma \equiv \sigma _0$, $h_{\mathrm {ext}}=0.1$, **b** $\sigma =\frac{\sigma _0}{\omega }$, $h_{\mathrm {ext}}=0.1$, **c** $\sigma \equiv \sigma _0$, $h_{\mathrm {ext}}=0.2$, **d** $\sigma =\frac{\sigma _0}{\omega }$, $h_{\mathrm {ext}}=0.2$

**Fig. 6:**
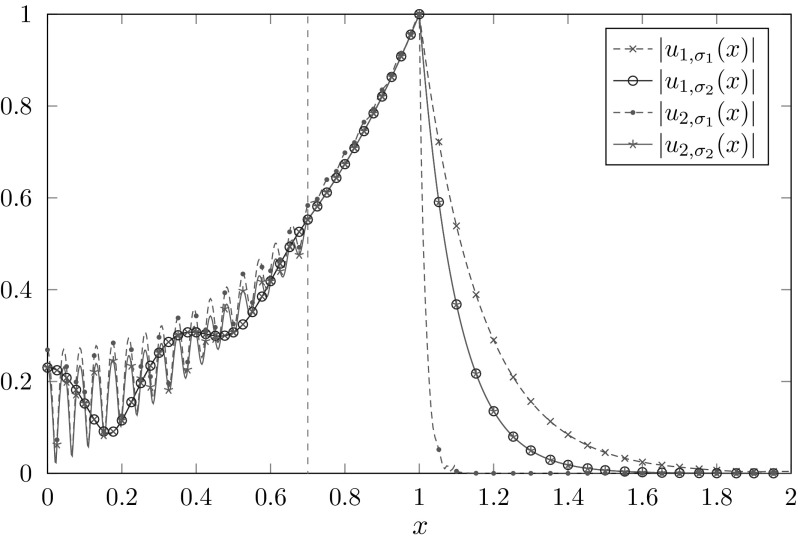
Absolute value of approximated eigenfunctions corresponding to $\omega _1\approx 8.1599{-}1.9769i$, $\omega _2\approx 65.2798{-}1.9769i$ with scaling functions $\sigma _1\left( \omega \right) \equiv 1+i$, $\sigma _2\left( \omega \right) =\frac{1+10i}{\omega }$

### Discretization of the truncated exterior domain

Using basis functions of order 10 in the interior domain and of order 5 in the exterior domain gives us a chance to study the effects of the discretization of the truncated exterior domain only (cf. Fig. 5). For the frequency independent complex scaling the exterior spurious resonances are located on a curve shaped similarly as the one containing the interior spurious resonances, but located much closer to the origin. At the intersection of this curve and the truncation resonances, the curve containing the latter exhibits a kink. This behavior has already been mentioned in [33] in the context of open elastic waveguides. The interior spurious resonances are no longer present since their location would be below the exterior spurious resonances.

A comparison of the eigenvalues for $\sigma _0=1+5i,1+10i$ in Figs. 4b, d and 5b, d leads to the conclusion that the exterior spurious resonances in the frequency scaled case are located on horizontal lines, similar to the truncation resonances. With increasing mesh-size $h_\mathrm{ext}$ they move farther away from the truncation resonances and closer to the real axis. This shift becomes larger for larger values of $\mathfrak {I}(\sigma _0)$ and larger mesh-size. For $\sigma _0=1+5i$ and $h_\mathrm{ext}=0.1,0.2$, as well as for $\sigma _0=1+10i$ and $h_\mathrm{ext}=0.1$ no exterior spurious resonances but again the truncation resonances can be observed.

Since the imaginary parts of the exterior spurious resonances are constant in all cases, they do not pollute the correct resonances of (2.1) near the real line. This dependency is less critical than the one in the case of the constant scaling, where fewer correct resonances can be computed using the same discretization.

In both the frequency dependent and the frequency independent complex scaling the location of the exterior spurious resonances depends only on the damping parameter $\sigma _0$, and the exterior mesh-size $h_{\mathrm {ext}}$. It is independent of the diameter of the complex scaled domain $T-R$. Because of (2.2), the absolute values of the resonance functions of (2.1) in the complex scaled domain are given by $\exp ( -\mathfrak {I}(\omega \sigma ) x)$. Thus, for the frequency dependent scaling $\sigma =\sigma _0/\omega$ the exponential decay is independent of the frequency. For the frequency independent scaling the resonance functions may decay too slow ($\mathfrak {I}(\omega \sigma _0)$ too small) resulting in large truncation errors or too fast ($\mathfrak {I}(\omega \sigma _0)$ too large) resulting in large approximation errors. The latter is the explanation for the worse results of the frequency independent scaling in Fig. 5a, c. Figure 6 illustrates the exponential increase and decay of the eigenfunctions corresponding to two resonances from Fig. 5a, b. Note that the computed resonance functions of the resonance with the smaller real part match perfectly in ${\varOmega _\mathrm{int}}$. The, ones corresponding to the resonance with the larger real part differ since this resonance already lies in a region, where the frequency independent scaling does not work properly any more.

### Full discretization and truncation

In the previous sections we legitimated the classification of spurious resonances into three categories: the discretization of the essential spectrum generated by the truncation (cf. Sect. 4.2), the interior spurious resonances (cf. Sects. 4.1 and 4.3) and the exterior spurious resonances (cf. Sect. 4.4). In all of our simulations, the interior spurious resonances are located on a curve connecting the negative imaginary axis with the positive real axis.

For the classical complex scaling with $\sigma \equiv \sigma _0$, the discretization of the essential spectrum is located on a straight line through the origin. The exterior spurious resonances lie on a similar curve as the interior ones but closer to the origin. For the frequency dependent version with $\sigma =\frac{\sigma _0}{\omega }$, the truncation resonances and the exterior spurious resonances are subsets of straight lines, parallel to the real axis. In all cases, true resonances can only be found in the smallest region bounded by the positive real axis, the negative imaginary axis, and the curves containing the truncation and the various spurious resonances. If, for a given set of parameters, resonances outside this region are sought, e.g. resonances near the real axis with larger real part, it is not a priori clear which parameters have to be adapted.

For the frequency dependent scaling the preceding results indicate that most often the discretization of the interior domain needs to be refined. Exterior spurious resonances near the real axis are only an issue, if the exterior discretization is very coarse in comparison to the interior one, or if $\mathfrak {I}(\sigma _0)$ is very small. For frequency independent scalings the situation is more involved. Figure 5 shows the possibility that the choice of $\sigma _0$ or of the exterior discretization is responsible for poor results. Thus, the frequency dependent scaling can be interpreted as an easy to use optimal choice of the exterior discretization, where the price to pay is the need to solve a polynomial eigenvalue problem.

## Effects of discretization in two dimensions

In this section we follow up our studies of the one-dimensional case in the previous section by analyzing two-dimensional examples. Since it is closely related to the Helmholtz equation in one-dimension, we start off with an example that can be simplified to the one-dimensional Bessel equation, followed by a two-dimensional example, where separation is not possible.

### Bessel equations

In the following, we consider Problem (2.8) with ${\varOmega _\mathrm{int}}:=B_R\left( 0\right) {\setminus } \overline{B_{R_0}\left( 0\right) }$, for $R>R_0>0$ and $p\left( \mathbf {x}\right) \equiv 1$. Using a separation ansatz, this problem simplifies to the well known Bessel equations. Applying a transformation $\tilde{u}_\nu \left( r\right) :=\sqrt{r+R_0}u_\nu \left( r\right)$ leads to the equivalent problem

5.1 $$\begin{aligned} -\tilde{u}_\nu ''\left( r\right) +\frac{\nu ^2-\frac{1}{4}}{\left( r+R_0\right) ^2}\tilde{u}_\nu \left( r\right) =\omega ^2\tilde{u}_\nu \left( r\right) , \end{aligned}$$

where homogeneous Neumann boundary values are transformed into Robin boundary conditions $\tilde{u}_\nu '\left( r\right) r+\frac{1}{2}\tilde{u}_\nu \left( r\right) =0.$ The resonances of (5.1) are the scaled roots of the first derivatives of the Hankel functions e.g. each resonance $\omega$ satisfies $\left( H^{(1)}_\nu \right) '\left( R_0 \omega \right) =0.$

**Fig. 7:**
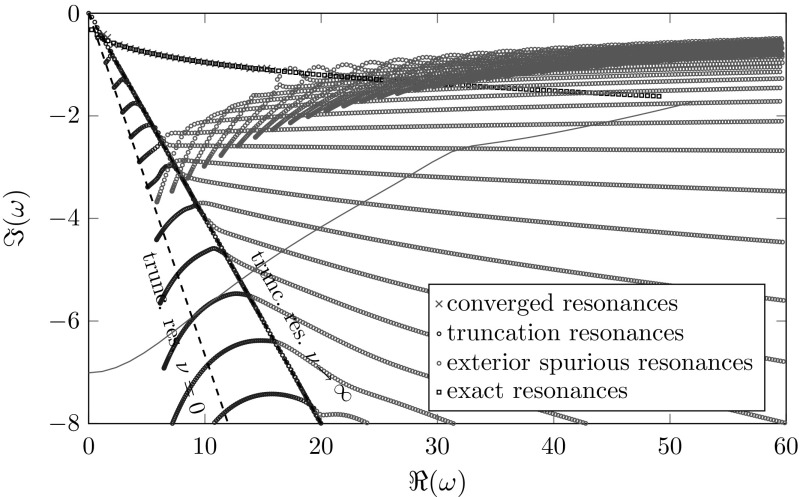
Approximated resonances of Bessel equations with indices $\nu =0,\ldots ,100$, $\varOmega _{\mathrm {int}}=(2,4)$, $\varOmega _{\mathrm {ext}}=(4,5)$, $\sigma \left( \omega \right) =1+2i$. The teal line marks the location of the interior spurious resonances from Sect. 4.1

**Fig. 8:**
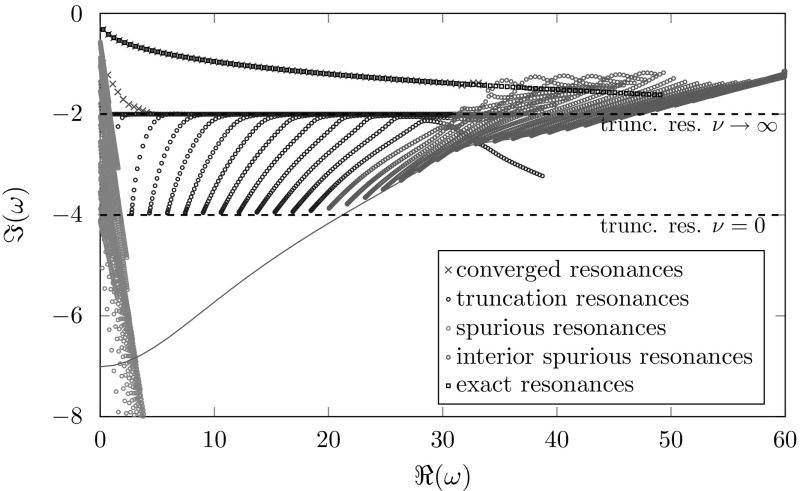
Approximated resonances of Bessel equations with indices $\nu =0,\ldots ,100$, $\varOmega _{\mathrm {int}}=(2,4)$, $\varOmega _{\mathrm {ext}}=(4,5)$, $\sigma \left( \omega \right) =\frac{10+8i}{\omega }$. The teal line marks the location of the interior spurious resonances from Sect. 4.1

The Bessel equations can be interpreted as a perturbation of the one-dimensional Helmholtz equation in the index $\nu$. Hence, we expect truncation resonances similar to the ones in the one-dimensional setting (4.4). As in Sect. 4, but with Hankel functions, the resonances of the truncated, complex scaled problem can be calculated by finding the roots of the determinant of a matrix resulting from the boundary values. In contrast to Sect. 4, no explicit expression of the truncation resonances is available. Nevertheless, semi-analytic calculations indicate a dependency of the truncation resonances on the index $\nu$, which can also be deduced from the discrete resonances given in Figs. 7 and 8. For these results we solved the resonance problem (5.1) with different indices using classical and frequency dependent complex scaling with the same mesh-size and polynomial order.

In the classical case we can observe truncation and exterior spurious resonances, similar to the one-dimensional case (cf. Sects. 4.2, 4.4) which depend also on the Bessel index $\nu$. The truncation resonances for a fixed index $\nu$ are still located on a straight line. For increasing index $\nu$ the angle of this line decreases and converges to some non-zero angle for $\nu \rightarrow \infty$. This behavior results in a sector of truncation resonances for multiple indices $\nu$ and is already mentioned in [25, Figs. 15 and 16] in the context of elastic waveguides. The more problematic exterior spurious resonances again behave like in the one-dimensional case for fixed $\nu$ and move closer to the real axis for larger indices $\nu$. The green line marks the location of the interior spurious resonances from Sect. 4.1, which are the resonances of (5.1) for $\nu =1/2$. Due to the location of the exterior spurious resonances no interior ones are calculated in this example.

For the classical complex scaling, resonances with absolute values up to around 16 are approximated properly for the chosen mesh-size, thickness of the damping layer and damping factor $\sigma _0$. In this case the limiting factor is the location of the exterior spurious resonances. Although these depend on the choice of the damping $\sigma =1+2i$ (confer with Fig. 5a), in all our computations using different damping factors the exterior spurious resonances stayed the limiting factor for the computations of the correct resonances. Thus, a finer discretization of the exterior domain and/or a different thickness of the damping layer is required to obtain better results.

In the case of the frequency dependent complex scaling we can observe the truncation/exterior and the interior spurious resonances already known from the one-dimensional case. Again for a fixed index $\nu$ both of them behave like in the one-dimensional case. For larger indices the truncation/exterior spurious resonances move upwards, but their imaginary part stays bounded away from zero resulting in a horizontal strip. Thus, the truncation/exterior spurious resonances do not interfere with the approximated correct resonances. The interior spurious resonances behave like the exterior spurious resonances in the classical case and move towards the real axis for growing $\nu$. Additionally, we can observe another set of spurious resonances located near the origin and the imaginary axis. Up to now the reason for these additional resonances remains unclear.

For the frequency dependent complex scaling all resonances with real parts up to around 32 are approximated properly. In this case the limiting factor is the location of the interior spurious resonances, which depends on the discretization of the interior domain. Therefore, in this case an optimization of the exterior discretization or the chosen complex scaling would not lead to any improvement.

### Full two-dimensional case

**Fig. 9:**
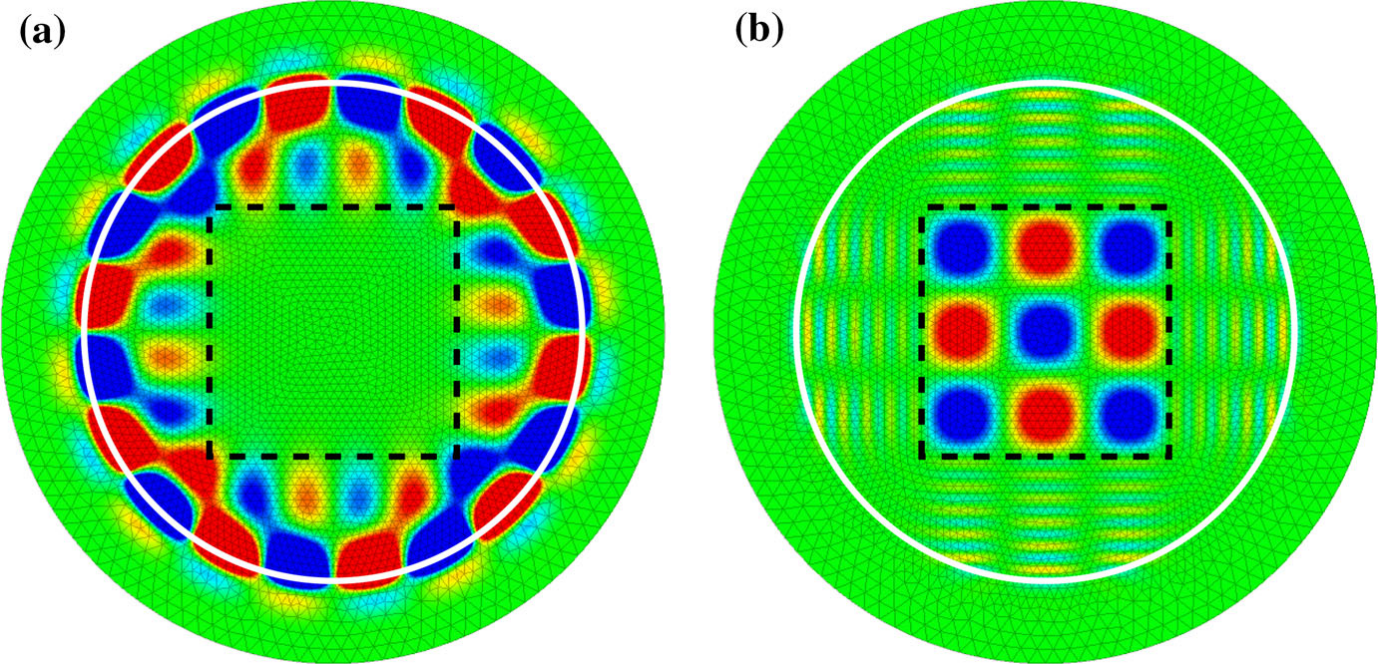
Real parts of resonance functions of the two-dimensional problem (cf. Fig. 11). The dashed square marks the jump in the potential, and the white circle the boundary between ${\varOmega _\mathrm{int}}$ and ${\varOmega _\mathrm{ext}}$. **a** $\omega \approx 4.90236{-}1.63113i$, **b** $\omega \approx 22.2143{-}0.67805i$

**Fig. 10:**
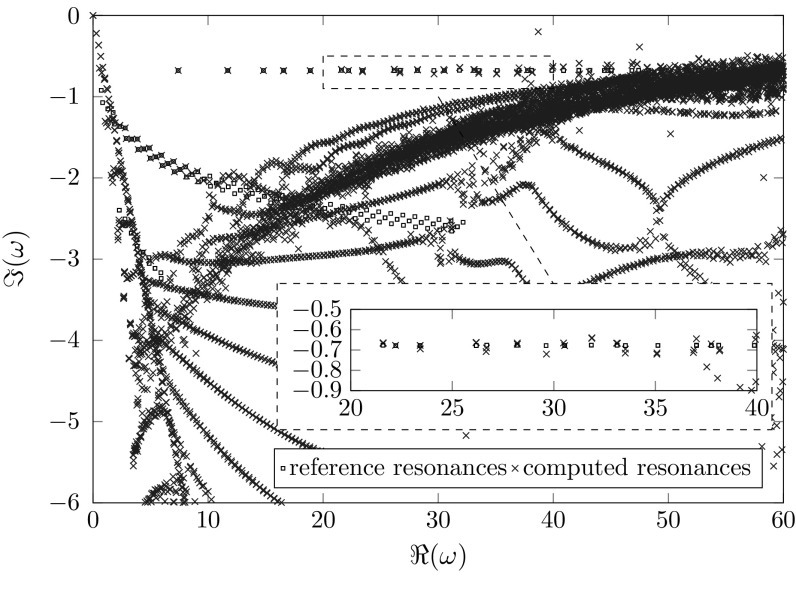
Computed resonances of a two-dimensional resonance problem using a classical complex scaling with $\sigma \left( \omega \right) \equiv 1+3i$, $h_\mathrm{{int}}=0.1,h_\mathrm{{ext}}=0.2$. The reference resonances were calculated using a higher polynomial order

**Fig. 11:**
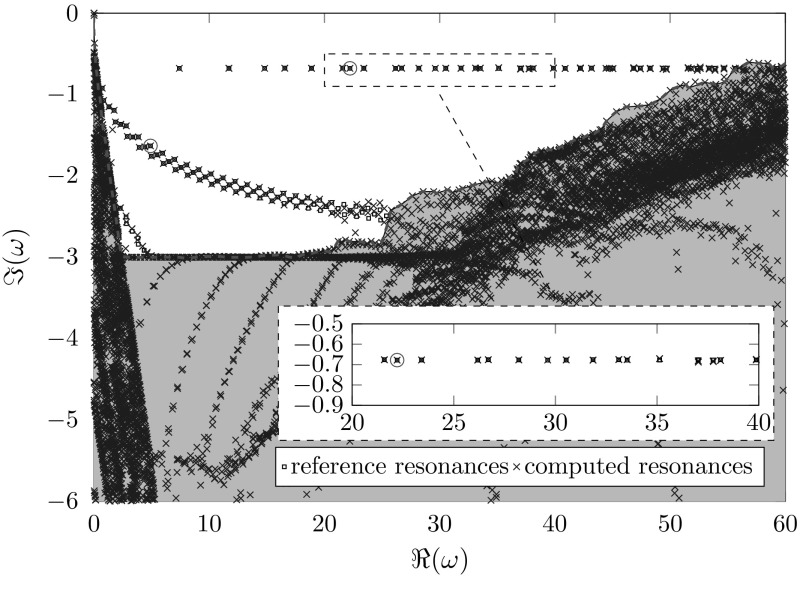
Computed resonances of a two-dimensional resonance problem using a frequency dependent scaling and $\sigma =\frac{9+9i}{\omega },h_\mathrm{{int}}=0.1,h_\mathrm{{ext}}=0.2$

Finally, we study a full two-dimensional problem using the high-order finite element implementation Netgen/NGSolve ([28, 29]). Due to the lack of rotational symmetry in this example no separation as in Sect. 5.1 is possible. We choose $\varOmega _{\mathrm {int}}:=B_{3}\left( 0\right)$, $\varOmega _{\mathrm {pml}}:=B_4\left( 0\right) {\setminus } \overline{B_3\left( 0\right) }$ and $p\left( \mathbf {x}\right) =1-0.8\chi _{[-2,2]^2}\left( \mathbf {x}\right)$. Figures 10 and 11 show resonances calculated using polynomial order 4, identical meshes, and a standard and a frequency dependent complex scaling respectively. We observe a kind of scattering resonances, which are perturbations of the roots of the Hankel functions and depend mainly on the volume $\partial [-2,2]^2$ of the “scatterer” (cf. Fig. 9a). Moreover we observe a set of transmission resonances with imaginary parts approximately − 0.7 (cf. Fig. 9b).

Focusing on spurious resonances, we observe similar effects as we did studying the Bessel equations. In the frequency independent case (Fig. 10) we see truncation and exterior spurious resonances. In the frequency dependent case (Fig. 11) we observe truncation and interior spurious resonances. Again, the additional artificial resonances near the imaginary axis can be observed. All of the aforementioned spurious resonances behave similarly as the ones of the Bessel equations in the frequency independent, as well as in the frequency dependent case.

**Fig. 12:**
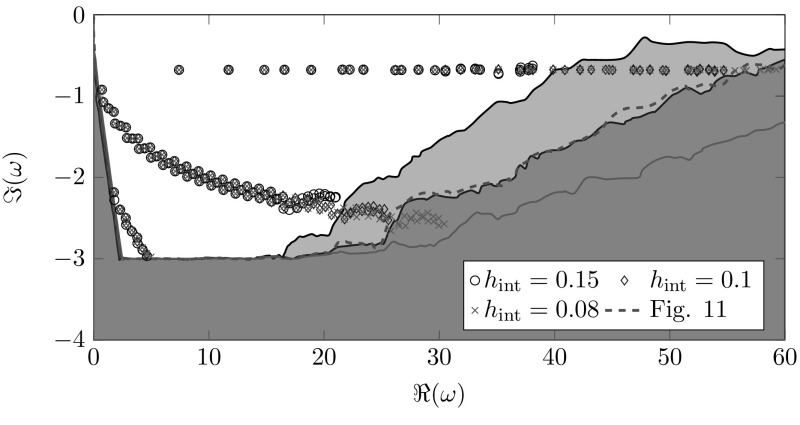
Location of spurious resonances for $h_\mathrm{{ext}}=0.15$ and different interior discretizations

For the frequency dependent scaling we study the effect of the meshsize on the location of the spurious resonances in Fig. 12. In order to enhance readability, only the correct resonances are plotted individually. Areas, which are polluted by artificial resonances are color-coded with respect to the interior meshsize. These areas tend towards the real axis for coarser interior discretizations. Note that the exterior meshsize used for Fig. 12 is finer as the one used for Fig. 11, but the location of the artificial resonances is the same for $h_\mathrm{{int}}=0.1$. This illustrates that the location of artificial resonances depends only on the interior discretization for the frequency depending scaling.

Overall, the frequency dependent complex scaling produces a larger quantity of correct resonances than the frequency independent complex scaling does since in the frequency independent case the exterior spurious resonances pollute the domain of interest. In principle, all remarks to the one-dimensional results remain valid for this two-dimensional problem as well.

## Conclusion and outlook

We have studied the effects of the various parameters and sub-steps in the discretization process of a complex scaling method on the computed resonances of the Helmholtz equation in one and two dimensions and have classified the occurring spurious resonances into three categories: truncation resonances, which are an approximation to the essential spectrum, and interior and exterior spurious resonances, generated by the discretization of the interior and exterior domain respectively. In applications where resonances near the real axis are sought-after, the interior and exterior spurious resonances are the ones raising problems, especially since the location of the exterior spurious resonances delicately depends on the choice of the damping parameter.

Moreover, we have in the context of resonance problems introduced and studied the use of a frequency dependent damping parameter resulting in a polynomial eigenvalue problem. In this case the same classes of spurious resonances appear, but take different shapes than in the frequency independent case. The exterior spurious resonances keep a constant distance to the real axis, since the exponential decay of the absolute value of the complex scaled resonance functions in the exterior domain does not depend as heavily on the frequency. Thus, resonances near the real axis are not polluted by exterior spurious resonances. Moreover, if the damping parameter is chosen within reasonable bounds, it has no impact on the number of well approximated correct resonances.

If solving the non-linear eigenvalue problems should be avoided, the frequency dependent scaling can still be used as a filter for the resonances computed with frequency independent scaling. Plugging the results of the linear eigenvalue problem into the discretization matrices of the frequency dependent scaling should lead to large condition numbers, if the result is the approximation of a resonance to the non-linear, frequency scaled resonance problem. So truncation and exterior spurious resonances can be detected by this procedure. Of course for this application a precise knowledge of the spectral properties of the non-linear eigenvalue problem resulting from frequency dependent complex scaling is crucial. In this way this paper might help to construct a reliable filter for spurious resonances as well.
